# Evolution of the lethality due to SARS-CoV-2 in Spain according to age group and sex

**DOI:** 10.1038/s41598-022-25635-y

**Published:** 2022-12-21

**Authors:** Pedro Saavedra, Angelo Santana, Alejandro Peñate, Carol Herrera, José-Miguel Pacheco

**Affiliations:** 1 Department of Mathematics, University of Las Palmas de Gran Canaria, 35017 Las Palmas de Gran Canaria, Spain; 2 Faculty of Health Sciences, University of Las Palmas de Gran Canaria, 35016 Las Palmas de Gran Canaria, Spain

**Keywords:** Viral infection, Statistical methods

## Abstract

The emergence of SARS-CoV-2 in China in December 2019 has posed a major challenge to health systems in all countries around the world. One of the most relevant epidemiological measures to consider during the course of a pandemic is the proportion of cases that eventually die from the disease (case fatality ratio, CFR). Monitoring the evolution of this indicator is of paramount importance because it allows for the assessment of both variations in the lethality of the virus and the effectiveness of the control measures implemented by health authorities. One of the problems with estimating the CFR in practice is that the available data only show daily or weekly counts of new cases and deaths; there is no information on when each deceased patient was infected and therefore it is not possible to know exactly how many cases there were at the time the patient became infected. Various approaches have been proposed for calculating the CFR by correcting for the time lag between infection and death. In this paper, we present a novel methodology to perform a non-parametric estimation of the evolution of the CFR by initially identifying an optimal time lag between infections and deaths. The goodness of this procedure is assessed by means of a simulation study and the method is applied to the estimation of the CFR in Spain in the period from July 2020 to March 2022.

## Introduction

At the beginning of March 2020, the severe acute respiratory syndrome coronavirus 2 (SARS-2-CoV-2) infection appeared in Spain and after a few weeks, in almost all of Europe. After a decrease in the number of cases detected in July of that year, there occurred successive waves of infections and deaths attributable to the virus. One question that is still not clear is how the lethality of the virus has evolved in the successive waves. There are many factors influencing this evolution^1,2^, especially vaccination campaigns and mutations in the virus genome that have changed its virulence and lethality over time. Although it now appears that the end of the pandemic is closer^3–5^, the immunity provided by vaccines decreases over time, while the risk of new variants evading immunity remains still as an uncertain factor. Therefore, even when the pandemic is declared over, health systems must remain vigilant^6^. In this sense, many countries have developed disease monitoring systems by constructing databases in which multiple variables are collected that provide information on patient characteristics—sex, age, comorbidities, date of onset, known exposures, etc. Efficient methods have been developed for the selection of variables^7–9^ that allow models to be developed to assess the risk of death and guide preventive policies.

During the course of a pandemic two main measures are used to assess the lethality of the virus^10^. The first is infection fatality ratio (IFR), which estimates the proportion of deaths among all infected individuals. The second is case fatality rate (CFR), which estimates the proportion of deaths among identified confirmed cases. The IFR is hard to estimate as it requires knowledge of the prevalence of the disease in the population, as well as the actual number of deaths that can be attributed to the disease^11^. While this number is readily available (at least in those countries with advanced health care systems), estimation of prevalence usually requires serological surveys, which are expensive and time-intensive^12^ and are therefore not suitable for rapid response to variations in virus lethality.

The case fatality ratio (CFR) is easier to obtain, but the fact that many asymptomatic or mildly symptomatic people are not identified as cases means that the CFR tends to overestimate the severity of the disease. However Luo et al.^13^ show that in locations that meet certain conditions (large-scale community transmission, extensive testing and without medical breakdown, as is the case of Spain) the CFR computed from cases detected by PCR (polymerase chain reaction) tests can be considered as a reliable indicator of the lethality of COVID-19. Traditionally, the fatality rate is assumed to be constant throughout the epidemic^14^, but this may not be true in rapidly evolving contexts^15–17^ such as the current SARS-CoV-2 pandemic. The simplest estimate of real time CFR is to divide the cumulative number of deaths by the cumulative number of cases over a period of time (usually 1 day or 1 week). This estimate is known as the crude (or naïve) CFR^18^. There are a number of reasons why this estimator may be biased^19,20^, but one of the most obvious is that the persons who die on a given day were infected much earlier, and thus the denominator of the CFR should be the total number of patients infected at the same time as those who died^21^. But usually the statistical records available show only daily or weekly numbers of new cases and deaths; there is no information on when each deceased patient was infected and therefore it is not possible to know exactly how many cases there were at the time the patient became infected.

A number of procedures have been proposed to circumvent this problem by correcting the CFR value to take into account the time elapsed between infection and death. Wilson et al.^22^ and Baud et al.^21^ estimated corrected CFR values for China during February 2020 by dividing the number of deaths on a given day by the number of patients with confirmed COVID-19 infection 13 or 14 days before. These values were chosen based on previous studies showing that the median time from infection to death was in this range. Shim et al.^23^ and Newall et al.^24^ compute a time delay adjusted CFR for COVID-19 in South Korea up to june 2020 fitting a gamma or lognormal distribution to the survival time of patients using previous case studies. Manisha et al.^25^ estimate the CFR for China and for countries outside China before April 2020 also using a 14-day delay between death and cases; but instead of simply dividing deaths by delayed cases, they also fit a regression line to these values and estimates the CFR as the slope of the regression, in an attempt to reduce the bias still present. Rothman et al.^26^ calculated age specific time corrected crude symptomatic CFR values from 7 countries using two independent time to fatality correction methods; Thomas et al.^18^ use what they call *time-shifted distribution (TSD) analysis method*. The CFR is obtained as the number of deaths in day $t$ divided by the number of cases in day $t-t_d$ where the value $t_d$ is obtained as the one that minimizes the root mean squared error in the linear regression of deaths versus delayed cases for $t_d=1,2,\dots ,25$.

In this paper a novel method is proposed to estimate the weekly progression of SARS-CoV-2 lethality (CFR) by age group and sex when available data are only daily total counts of cases and deaths. Like Thomas et al.^18^ we propose to approximate the number of deaths occurring among the cases registered in the week $w$ by the number of deaths that occur $\delta$ weeks later. The CFR is then estimated by using a log-additive model^27^ selecting for $\delta$ the value which minimizes the Akaike Information Criterion^28^. The goodness of this procedure is assessed by means of a simulation study based on actual data. The method is applied to the estimation of the CFR in Spain in the period from July 2020 to March 2022.

## Material and methods

### Data source

All data has been obtained from the Carlos III Health Institute (ISCIII)^29^. This institute is the Public Research Organization of the Spanish Government responsible for funding and executing national biomedical research and depends on the Ministry of Science, Innovation and Universities of Spain, and it is also attached to the Ministry of Health, Consumption and Social Welfare. This institution maintains a panel of open data obtained through the epidemiological surveillance network of the National Epidemiology Centre, which can be downloaded from its website^30^. The data available are daily counts of reported COVID-19 cases, number of deaths, number of people hospitalised and number admitted to ICU, broken down by age group and sex.

Data on the number of infections in the first wave of the pandemic are not reliable as only highly symptomatic cases were detected at that time^31^. For this reason, in this study only data from week 20–26 July 2020 to week 14–20 March 2022 (87 weeks) have been considered. The study was carried out separately for three age groups, namely: *“less than 50 years”*, *“from 50 to 69 years”* and *“70 or over years”*. For each of these groups, the data used in the analysis are the weekly counts of infections and deaths by sex group. More specifically, they are of the form:

$$\begin{aligned} \begin{aligned} \left\{ \left( \textit{Cases}_{\textit{Sex},w}, \textit{Deaths}_{\text {Sex},w}\right) : \text {Sex}=Male,Female;w=1,\ldots ,87\right\} \end{aligned} \end{aligned}$$

where $\textit{Cases}_{\text {Sex},w}$ and $\textit{Deaths}_{\text {Sex},w}$ denote the count of registered SARS-CoV-2 cases and deaths by sex group and week.

**Figure 1:**
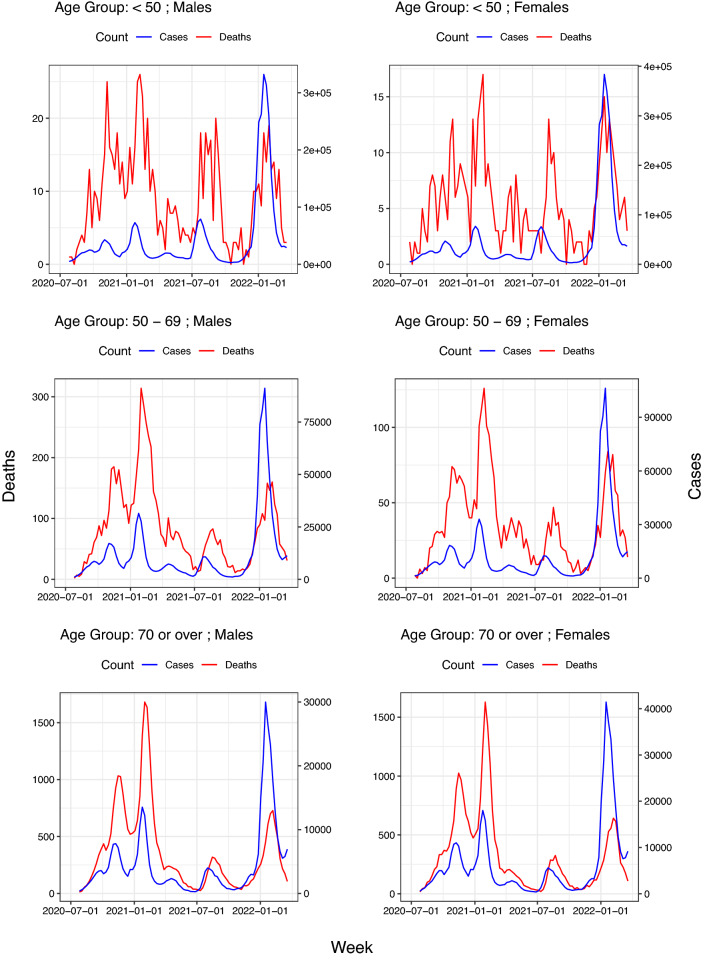
Joint plot of weekly cases and deaths counts by age group and sex. Note that the trajectories of the counts of deaths appear shifted to the right with respect to that of the cases and that such displacements depend on age group. The strong oscillation of the death counts in the group under 50 years of age is attributable to the low lethality rate since it increases the coefficient of variation of deaths.

### Statistical analysis

For each age group, we denote by $d_{\text {Sex},w}$ the *count of deaths among those subjects registered as SARS-CoV-2 cases at week* $w$. These deaths will therefore occur over the next few weeks. We assume that:

1 $$\begin{aligned} {d_{\text {Sex},w}\sim \text {binomial}\left( \text {Cases}_{\text {Sex}, w};p_{\text {Sex}}(w)\right) } \end{aligned}$$

where $p_{\text {Sex}}\left( w\right)$ is the *actual Case Fatality Rate* at $w$, i.e. is the probability of dying for those subjects registered as SARS-CoV-2 cases in week $w$.

In addition, we assume for $p_{\text {Sex}}\left( w\right)$ the additive log-model^27^:

2 $$\begin{aligned} {\mathrm {\log }\left( p_{\text {Sex}}(w)\right) =\alpha +\beta _{\text {Sex}}+s\left( w\right) } \end{aligned}$$

Here, “log” denotes the natural logarithm, $\beta _{\text {Female}}=0$ (Female sex is taken as reference) and $s\left( w\right)$ is a *smooth* function of the week, which will be estimated nonparametrically using cubic splines.

The ISCIII data do not provide the values of $d_{\text {Sex},w}$ and, therefore, the CFR $p_{\text {Sex}}\left( w\right)$ cannot be directly estimated. However, Fig. 1 suggests that for each sex group there is a proportionality between the case count at week $w$ ($\textit{Cases}_{\text {Sex},w}$) and that of deaths $\delta$ weeks later ($\textit{Deaths}_{\text {Sex},w+ \delta }$) for some delay $\delta$, leading to the fact that:

3 $$\begin{aligned} { Death_{Sex,w+\delta }\sim binomial\left( Cases_{Sex,w}; {\widetilde{p}}_{Sex}\left( w\right) \right) } \end{aligned}$$

Here we assume that $\delta$ depends on sex but not on $w$. This parameter could be considered as the expected survival time of those cases who die.

In order to determine the delay $\delta$ by sex group, we fit the model (3) for values $\delta \in \left\{ 0,1,2,3,4,5\right\}$ estimating ${\widetilde{p}}_{Sex}\left( w\right)$ nonparametrically by means of a cubic spline. For each $\delta$ the *Akaike Information Criterion*^28^ $AIC\left( \delta \right)$ is computed which measures the lack of fit of the model. Finally, we consider as optimum the value of $\delta$ that minimizes $AIC\left( \delta \right)$. Note that the value of $\delta$ obtained depends on the sex group.

We can expect that most of the deaths among the cases registered in week $w$ will occur on the days of week $w+\delta$. There will certainly be deaths among such cases occurring outside that period. Similarly, some of the deaths corresponding to cases registered in weeks close to $w$ will occur in week $w+\delta$. If we assume that $p_{\text {Sex}}\left( w\right)$ varies smoothly from 1 week to the next, then cases registered in $w$ that die outside $w+\delta$ are roughly offset by deaths in $w+\delta$ corresponding to cases registered outside but close to $w$. Thus the smooth variation assumption of $p_{\text {Sex}}\left( w\right)$ leads us to the approximation: $d_{Sex,w}\approx Death _{Sex,w+\delta }$, and therefore the CFR $p_{\text {Sex}}\left( w\right)$ in the log-additive model (2) can be estimated by ${\widetilde{p}}_{Sex}\left( w\right)$ in model (3). We examine the goodness-of-fit of this method by a simulation study in the next section.

Once the models given in (1) are estimated for each age group, the rate ratios *Males:Females* (*RR*) are obtained as $\exp \left( \beta _{M}\right)$ and estimated by means of 95% confidence intervals (CI). Progression of the CFR is expressed as *expected deaths by 10,000 infections-week* $\left( 10^{4}\times p_{\text {Sex},w}\right)$.

Statistical significance was set at $p<0.05$. Data were analyzed using the R statistical language and environment, version 4.2.1^32^. 

## Simulation study

As input for this study, the actual data of the number of cases corresponding to the cohort of males aged 70 years or older have been used. The probability of death among those registered at week *w* (*theoretical CFR*) has been modelled using a function $p(w)$ with two peaks of lethality. This function is shown in Fig. 3.

**Figure 2:**
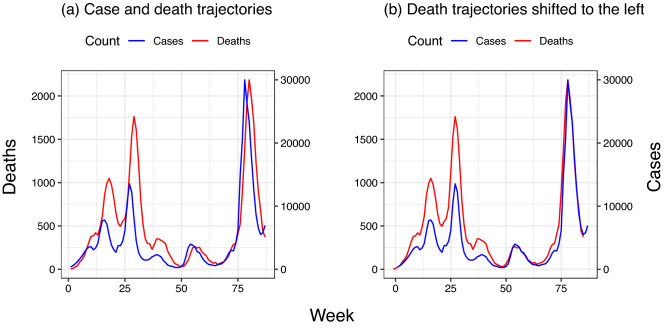
Simulated trajectories of cases and deaths. The expected time of survival among the death was 2 weeks and thus, the trajectory of death was delayed 2 weeks.

The simulation proceeds as follows:

1. As the number of new cases in week $w$ ($\text {Cases}_{w}$) we use the number that actually occurred in Spain between 20–26 July 2020 and the week 14–20 March 2022 (87 weeks).
2. For week $w$ in which $\text {Cases}_{w}$ infections have been recorded, the number of those who will die, $d_{w}$ is generated from a distribution $binomial\left( \text {Cases}_{w},p(w)\right)$.
3. Survival times for each patient are generated independently with a common Weibull probability distribution with parameters $\lambda =2$ and $\kappa =2.257$. Thus, the expected survival time among those who are going to die is 2 weeks (the estimate for actual data is 1 week, but here we use 2 weeks because this increases the variance in survival time and spreads the number of deaths over longer periods, making the CFR more difficult to estimate).
4. Figure 2a shows the weekly counts of infections ($\text {Cases}_{w}$) and the count of deaths ($\text {Deaths}_{w}$) obtained in the simulation. Figure 2b show the same data but with deaths delayed by 2 weeks.
5. For values of $\delta \in \left\{ 0,1,2,3,4,5\right\}$, we estimate the model $\text {Deaths}_{w+\delta }\sim \text {binomial}\left( \text {Cases}_{w},p(w)\right)$, being $p(w)=\alpha +s(w)$ (we are considering only males) . $\text {AIC}\left( \delta \right)$ denotes the corresponding AIC value. Note that the optimal $\delta$ coincides with the expected survival time (Weibull distribution).

**Figure 3:**
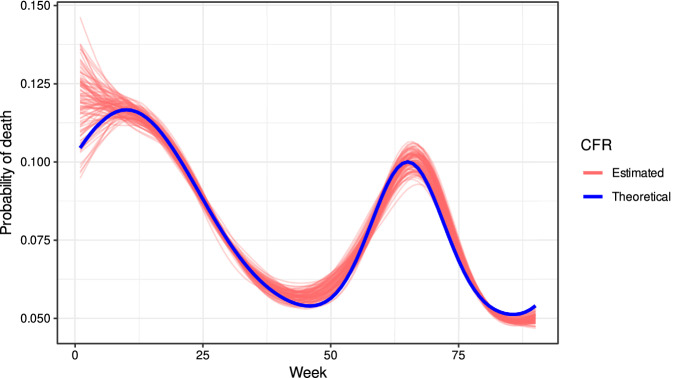
Simulation study: lethality rate (theoretical and estimated in 100 simulations).

Figure 3 plots the CFR estimated from these data in 100 simulations of the process, as well as the theoretical CFR, and shows that the procedure described produces a good estimate of the CFR used to simulate the data.

## Results

**Figure 4:**
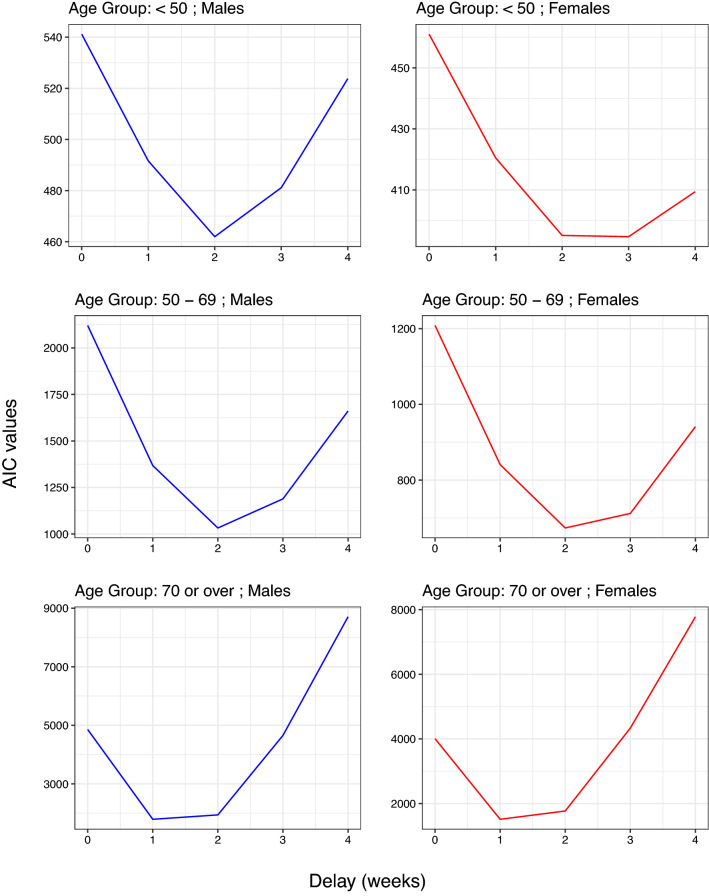
Values of AIC according to delay by age group and sex.

Table 1 summarizes the six cohorts of study (three age groups for each sex). For the entire follow-up period and the three age groups, the infection rates were similar by sex, but mortality rates were higher in men than in women. Figure 1 displays jointly the weekly counts of *cases* and *deaths* (note the different scales on the y-axis) for each age group and sex. Note that the trajectories of deaths exhibit a shift to the right relative to that of infections, which appears to be week independent. Such displacements would correspond to the *expected times of survival* among those patients who die. Figure 4 shows the values of AIC versus the different delays corresponding to model (3) and their corresponding optima $\delta$.

**Table 1:** Summary of the study cohorts for the period July 2020–March 2022.

Age (years)	Gender	Population	Infections		Deaths	
			Count	Rate	Count	Rate
$<50$	Male	14,213,918	3,748,502	263.7	799	56.21
	Female	13,791,776	4,102,042	297.4	489	35.46
50–69	Male	6,124,088	1,053,728	172.1	7288	1190
	Female	6,384,636	1,168,291	183	3126	489.6
70 or over	Male	2,870,781	401,664	139.9	31,437	10,951
	Female	3,970,486	548,416	138.1	28,257	7117

Infection rate is shown as number of infected per 1000 people and mortality rate is calculated as number of deaths per million people in the population.

**Figure 5:**
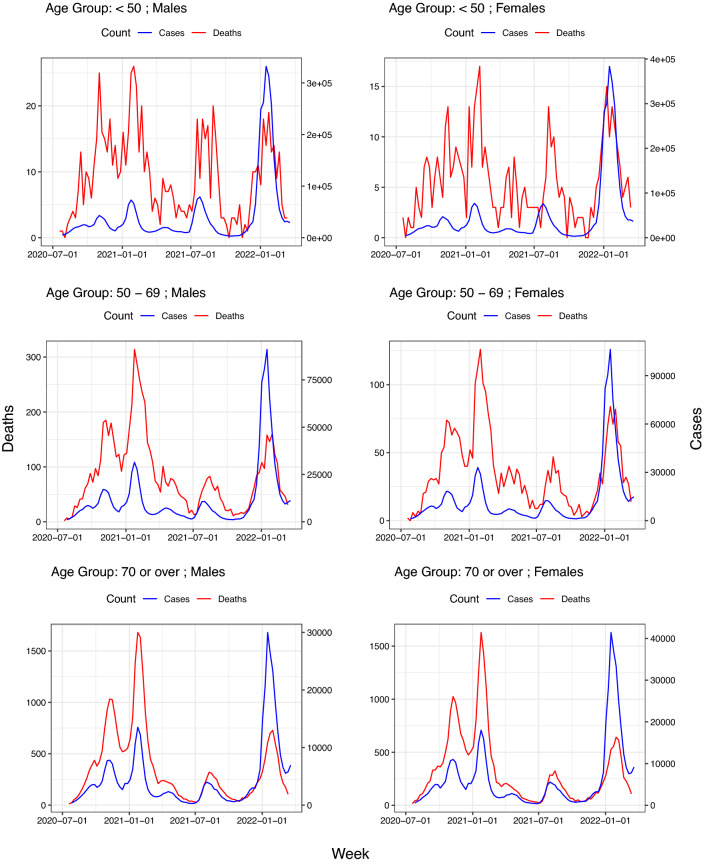
Trajectories of the weekly counts of cases and deaths. When the death curves are shifted to the left the corresponding number of weeks, there tends to be a proportionality between infections and deaths; the divergences correspond to variations in the lethality rates.

**Figure 6:**
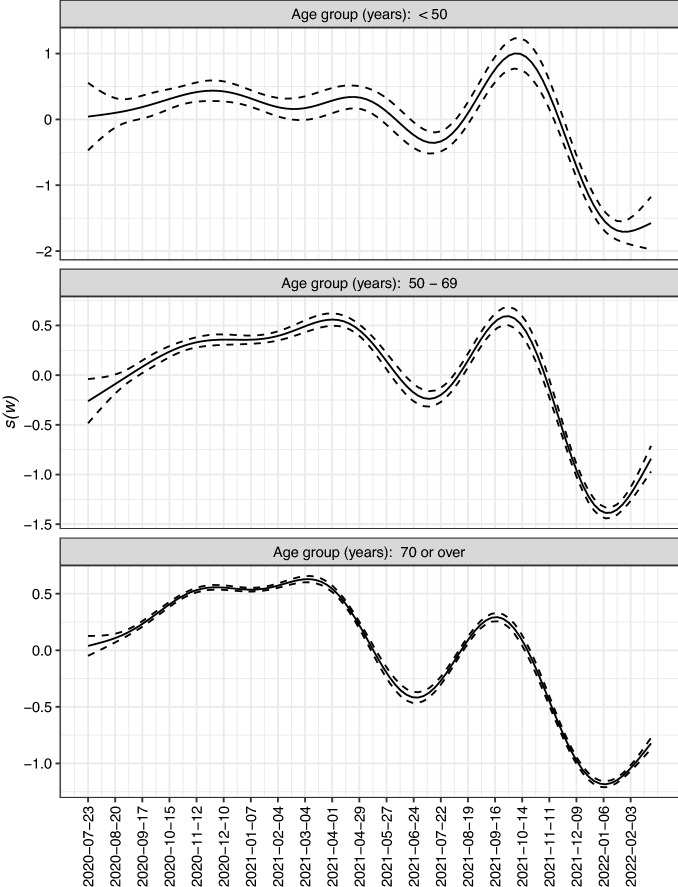
Splines corresponding to the progress of the lethality rate (95% CI) according to age group.

**Figure 7:**
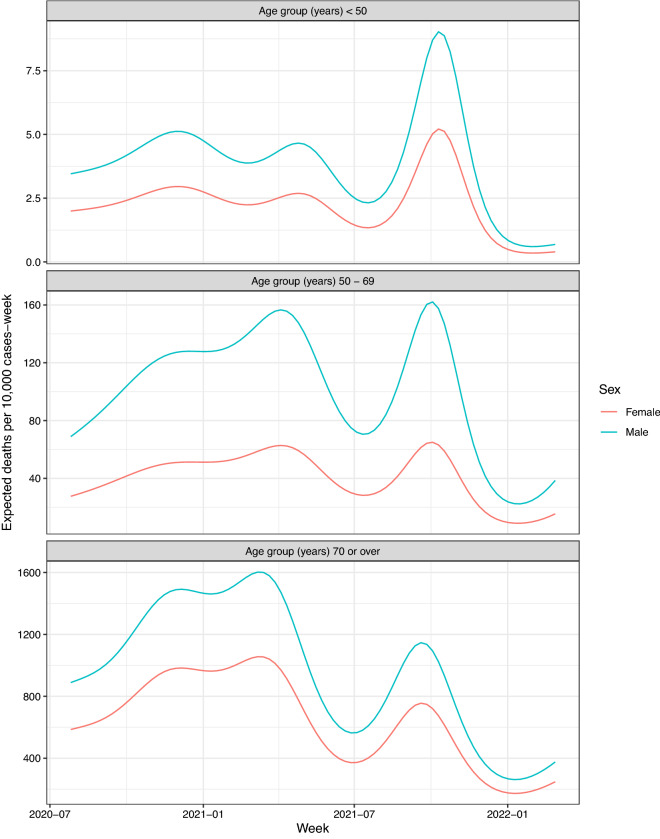
Progress in the lethality rate by age group and sex.

Note that the older the patients, the lower the $\delta$ values. When the trajectories $\text {Cases}_{\text {Sex},w}$ and the approximations of $d_{\text {Sex},w}$ by $\textit{Death}_{Sex,w+\delta }$ are plotted together (Fig. 5), it can be observed that they show to be *in phase*. Table 2 summarizes the estimation of the three binomial models, one for each age group. The estimated $s(w)$ curves in the additive models (2) are shown in Fig. 6. It can be observed that for each of the three age groups three peaks were reached during the follow-up period. In addition, Fig. 7 displays the progress in the CFR rate (expressed as expected deaths by 10,000 persons-week) for each age group showing also the differences by sex as estimated by the model. In all groups, the CFR was significantly higher in men. For the group of patients under 50 years of age, the CFR rate ratio of men vs. women was *RR* = 1.73 (95% CI 1.55; 1.94); for those between the ages of 50 and 69, the rate ratio was the highest *(RR* = 2.49: 95% CI 2.39; 2.60). Finally, for the group of 70 years or older, it was found that the CFR rate was 50% higher in men than in women (*RR* = 1.52: 95% CI 1.49; 1.54).

**Table 2:** Estimation of the binomial models by age group.

Age group (years)	Factor	Coefficient (SE)	p value	Rate ratio (95% CI)
$< 50$	Intercept ($\alpha )$	$-$ 8.562 (0.049)	$< 0.001$	
	*Sex*			
	Female (Ref.)	0	–	1
	Male ($\beta _{M})$	0.549 (0.058)	$< 0.001$	1.73 (1.55; 1.94)
	*Week*	Cubic spline	$< 0.001$	
50$-$ 69	Intercept ($\alpha )$	$-$ 5.630 (0.020)	$< 0.001$	
	*Sex*			
	Female (Ref.)	0	–	1
	Male ($\beta _{M})$	0.914 (0.021)	$< 0.001$	2.49 (2.39; 2.60)
	*Week*	Cubic spline	$< 0.001$	
70 or over	Intercept ($\alpha )$	$-$ 2.876 (0.007)	$< 0.001$	
	*Sex*			
	Female (Ref.)	0	–	1
	Male ($\beta _{M})$	0.417 (0.008)	$< 0.001$	1.52 (1.49; 1.54)
	*Week*	Cubic spline	$< 0.001$	

## Discussion

**Figure 8:**
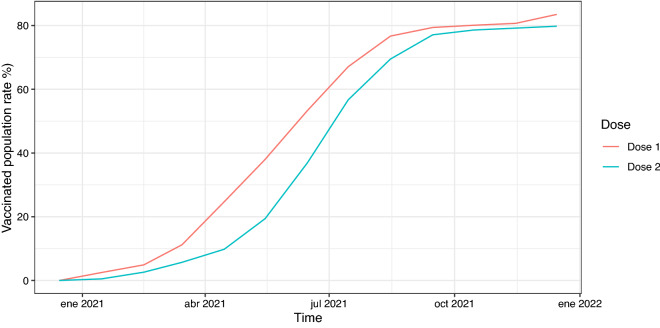
Progression of vaccination Rates in Spain.

The results obtained show how SARS-CoV-2 case fatality ratio has evolved during the course of the pandemic. Once Spain reached a stage of stable extensive testing, this progression of lethality rates could be explained by new virus variants that emerged during the observation period (see Table 3), as well as by the gradual introduction of vaccination against SARS-CoV-2 from the beginning of 2021 (see Fig. 8).

**Table 3:** Dates and places of first detection of new virus variants.

WHO level	PANGOLIN lineage	Detection	First case sequenced in Spain
Alpha	B.1.1.7	September 2020	December 2020
		United Kingdom	
Beta	B.1.351	May 2020	December 2020
		South Africa	
Gamma	P.1	November 2020	January 2021
		Brazil	
Delta	B.1.617.2	October 2020	April 2021
		India	
Omicron	B.1.1.529	November 2021	November 2021
		South Africa	

Figure 7 shows that CFR increases with age and is higher in men than in women for all age groups. These results are in line with those of^33^, who studies the lethality in the first wave of SARS-CoV-2 in Spain. This sex disparity is likely explained by a combination of biological sex differences (differences in chromosomes and related sex steroids) and gender-specific factors such as differential behaviors. Men are more likely to engage in poor health behaviors such as smoking and alcohol consumption resulting in higher rates of pre-existing co-morbidities (hypertension, cardiovascular disease, COPD) associated with a poor COVID 19 prognosis^34^. Concerning the smoking habit, current smokers compared with never smokers have significantly upregulated ACE2 expression in the oral and lung epithelium. Given that smoking is more prevalent among males, higher expression of ACE2 due to this risk factor could explain the worse outcomes of SARS-CoV-2 in males^35,36^. Additionally, women have a higher antibody production and more efficacious vaccine responses overall. Furthermore, healthy females are known to have higher numbers of CD4+ T cells, greater CD4+/CD8+ ratios and an increased number of activated T cells, cytotoxic T cells, and B cells compared with males, resulting in a prompter response to the presence of infection^34,37^. Finally, low levels of testosterone in elderly men have been associated with upregulation of inflammatory markers and possible risk of lung damage, as well as respiratory muscle catabolism and increased need for assisted ventilation^38^. All of these factors could contribute to a poorer response to SARS-CoV-2 infection in men, but this requires further research.

### Limitations and future work

The procedure described in this article estimates the CFR at the week $w$ by assuming that for each sex and age group the number of deaths at $w+\delta$ follows a $binomial\left( Cases_{w};{\widetilde{p}}\left( w\right) \right)$ distribution with ${\widetilde{p}}\left( w\right)$ similar to $p\left( w\right)$, the true CFR. This approximation is valid when both the variation in the number of cases and the evolution of virus lethality occur smoothly. When a peak occurs in the number of cases the approximation does not work well. It can be seen in the simulation (Fig. 3) how the estimation of the function $p\left( w\right)$ is worse around week 70, when there was a peak in the number of cases caused by the irruption of the omicron variant. Further refinements of this work are related with the fact that the CFR may have other biases^19,20^ that we have not corrected for. Future work is required to improve the approximation at times when there is a rapid increase in the number of cases or mortality and to introduce corrections for other biases. It is also interesting to study how this CFR estimate can be used to improve estimates of the IFR, the other important lethality index, which is extensively used by health systems for pandemic management.

## Conclusion

The procedure we have developed in this paper to estimate the CFR during a pandemic aims to correct for the bias due to the time lag between the time of infection and the time of death. Previous works make this correction by calculating the CFR as the number of deaths at $t+\delta$ divided by the number of cases at $t$. In those papers, several strategies are followed to estimate the appropriate value of $\delta$: using $\delta$ values chosen subjectively by the researcher, using clinical data to estimate the distribution of survival time, or using the similarity of the curves of cases $C(t)$ and deaths $D(t)$ to determine the value of $\delta$ for which a better linear fit is achieved $D(t+\delta )=\lambda C(t)$. Our procedure improves this approximation by considering that $D(t+\delta )$ follows for each sex and age group a binomial distribution of parameters $C(t)$ and $p(t)$, where $p(t)$ is estimated non-parametrically by means of a spline that allows us to directly obtain the evolution of the CFR. This procedure could be promising for decision-making in public health policies in a scenario in which the vaccination rate remains constant in a high percentage of the population, and changes in the CFR could basically be attributed to changes in virus lethality.

## Data Availability

The dataset supporting the conclusions of this article is publicly available through the web page of the Carlos III Health Institute (Spain), https://cnecovid.isciii.es/covid19/#documentación-y-datos. R source code for the analysis is available at www.github.com/angeloSdP/covidWeek.

## Abbreviations

AIC: Akaike information criteria
CFR: Case fatality ratio
IFR: Infection fatality ratio
ISCIII: Instituto de Salud Carlos III (Carlos III Health Institute)
RR: Rate ratio
